# Beneficial effects of Silexan on co-occurring depressive symptoms in patients with subthreshold anxiety and anxiety disorders: randomized, placebo-controlled trials revisited

**DOI:** 10.1007/s00406-022-01390-z

**Published:** 2022-03-09

**Authors:** Lucie Bartova, Markus Dold, Hans-Peter Volz, Erich Seifritz, Hans-Jürgen Möller, Siegfried Kasper

**Affiliations:** 1 Department of Psychiatry and Psychotherapy, Medical University of Vienna, Währinger Gürtel 18–20, 1090 Vienna, Austria; 2 Hospital for Psychiatry, Psychotherapy and Psychosomatic Medicine, Schloss Werneck, Balthasar-Neumann-Platz 1, 97440 Werneck, Germany; 3 Department of Psychiatry, Psychotherapy, and Psychosomatics, Psychiatric Hospital, University of Zürich, Lenggstrasse 31, 8032 Zürich, Switzerland; 4 Clinic and Policlinic for Psychiatry and Psychotherapy, Ludwig-Maximilian-University Munich, Nußbaumstraße 7, 80336 Munich, Germany; 5 Center for Brain Research, Medical University of Vienna, Spitalgasse 4, 1090 Vienna, Austria

**Keywords:** Anxiety disorders, Subthreshold anxiety, Depression, Effects, Lavender, Meta-analysis, Silexan

## Abstract

Silexan is a proprietary active substance produced from *Lavandula angustifolia*, with proven anxiolytic efficacy in subthreshold and generalized anxiety disorder as well as in mixed anxiety and depressive disorder with beneficial impact on anxiety-related sleep disturbances. The pharmacological profile and clinical observations suggest that Silexan may also have an antidepressant effect. To investigate the effect of Silexan on co-occurring depressive symptoms, we present a meta-analysis of the five placebo-controlled clinical trials hitherto performed with Silexan in subthreshold anxiety (*n* = 3) and anxiety disorders (*n* = 2). Patients of all trials received Silexan 1 × 80 mg/day or placebo for 10 weeks according to random assignment. Assessment of the antidepressant effect was based on item ‘depressed mood’ from the Hamilton Anxiety Rating Scale (HAMA) administered in all trials and on the total scores of the Montgomery Åsberg Depression Rating Scale (MADRS) or the Hamilton Depression Rating Scale (HAMD) used in three trials. After 10-week treatment, patients receiving Silexan showed significantly more pronounced score reduction for HAMA item ‘depressed mood’ than those in the placebo group (*p* = 0.01). Significant superiority of Silexan over placebo could also be shown for mean MADRS or HAMD total score reduction (three studies; *p* < 0.01). Silexan-treated patients with more severe depressive symptoms at baseline showed more pronounced improvements than those with milder symptoms. Our meta-analysis clearly shows that Silexan has a beneficial effect on co-occurring depressive symptoms in patients with subthreshold anxiety and anxiety disorders and may, hence, lead to important therapeutic implications for depressive disorders.

## Introduction

Anxiety disorders and major depression are the most prevalent mental ilnesses, accounting for more than half of the disease burden attributable to psychiatric diseases worldwide [1]. For anxiety and mood disorders, a meta-analysis based on 85 surveys covering more than 60 countries found lifetime prevalences of 12.9% and 9.6% as well as 12-month prevalences of 6.7% and 5.4%, respectively [2]. In Europe and the United States, these figures are even higher. In large epidemiological studies, 12-month prevalences were 14% and 18%, respectively, for anxiety disorders as well as 7.8% and 9.5%, respectively, for mood disorders [3–5].

Clinical experience as well as empirical data indicate that anxiety and depression are highly comorbid [6, 7]. Moreover, anxiety has been shown to predict later depression and vice versa, both on an individual symptom level and on the disorder level [8]. It has been estimated that up to 90% of patients with an anxiety disorder also exhibit symptoms of depression [9], and between 30 and 63% also meet the criteria for concurrent major depressive episode (MDE) [10]. Among the most central symptoms in both depression and anxiety are anhedonia, sad mood, and worry [11]. Due to symptom overlap, it is not surprising that the Hamilton Anxiety Rating Scale (HAMA) [12] includes items that assess depressed mood as well as symptoms overlapping with major depressive disorder (MDD, e.g., concentration), while the Hamilton Depression Rating Scale (HAMD) [13] includes items that assess anxiety.

Comorbid anxiety and depression are typically associated with a more severe clinical presentation than either condition alone [14], including greater severity and longer duration of illness, more severe functional impairment, and ultimately poorer clinical outcomes [15]. Patients with comorbid anxiety and depression were found to be more treatment resistant than those with either condition alone [7, 10, 16–19]. Moreover, it has been observed that co-morbidity of anxiety and depression increases the risk of exacerbation, e.g., patients suffering from subthreshold anxiety disorder with co-morbid depressive symptoms or with mixed anxiety and depressive disorder (MADD) may be at an increased risk of progressing to generalized anxiety disorder (GAD) or to MDD [9, 20]. The observation that subthreshold anxiety often constitutes a predictor of subsequent GAD or MDD is, therefore, of great value for prevention and may have important implications for treatment [21].

Nonclinical data indicate that there may be common neurobiological pathways to both anxiety and depression, most notably a dysregulation of the norepinephrine and serotonin (5-HT) neurotransmitter systems [22]. An increased neurotransmitter-release due to an enhanced Ca2^+−^influx mainly through N- and P/Q-type voltage dependent calcium channels (VDCCs) [23] and variations in serotonin-1A (5-HT_1A_) receptor binding [24, 25] may play a role in both types of disorder.

The interpretation is supported by the fact that substances with proven efficacy in the treatment of depression have been demonstrated to be efficacious in anxiety disorders as well. This is particularly true for selective serotonin reuptake inhibitors (SSRIs), whose efficacy in anxiety and depression has been linked to their agonistic action on the 5-HT_1A_ receptor subtype [26, 27]. Consequently, agents such as SSRIs and selective norepinephrine reuptake inhibitors (SNRIs) that were originally developed as antidepressants are also recommended as first line treatment for anxiety disorders (e.g., [28]). There also appears to be a growing interest in the anxiolytic and antidepressant effects of preparations from lavender, with six reviews and meta-analyses published during 2019 and 2020 alone [29–34].

For Silexan,^1^ an essential oil for oral administration manufactured from *Lavandula angustifolia* flowers, a potent inhibition of VDCCs in synaptosomes, primary hippocampal neurons and stably overexpressing cell lines [35], attenuating the overreaching, situationally inadequate stress response of the central nervous system associated with anxiety and mood disorders has been assumed (e.g., [36]). The active substance was shown to significantly increase the density of 5-HT_1A_ receptors and to reduce the serotonin-1A receptor binding potential, leading to increases in extracellular serotonin, dopamine, and norepinephrine [37, 38]. A comprehensive characterization of the pharmacological profile of Silexan has been provided elsewhere [39, 40].

Silexan is the active substance of a medicinal product used for the treatment of anxiety. Treatment with Silexan was shown to be safe, without causing pharmacological interactions, sedation, or withdrawal symptoms at daily doses of 80 or 160 mg [39]. Randomized, double-blind, controlled clinical trials have demonstrated that Silexan has a significant anxiolytic effect in subthreshold anxiety disorder, MADD, and GAD [31, 41]. Results from these trials indicate that Silexan may also have an antidepressant effect [42] which could be explained by its impact on serotonergic mechanisms typically observed for serotonergic substances as SSRIs and SNRIs for instance [37, 38, 40, 43]. This might be of relevance especially in terms of its beneficial effects on sleep disturbances that rank among the most common and burdensome symptoms in both anxiety and depressive disorders [44]. In a retrospective case series on the use of Silexan in patients suffering from MDD and symptoms of psychomotor agitation, insomnia and anxiety, a reduction of anxiety-related symptoms and sleep disturbances, psychological anxiety and somatic anxiety was observed [45]. In addition, results from a recently published meta-analysis investigating all existing placebo-controlled clinical trials in anxiety patients treated with Silexan revealed statistically significant and clinically meaningful effects of Silexan over placebo in improving somatic symptoms as insomnia, fatigue and pain, which count to frequently occurring symptoms of both, anxiety and depressive disorders [46].

While compounds originally developed as antidepressants have been shown to be efficacious in the treatment of anxiety disorders as well, it might, therefore, be promising to assess the potential of Silexan, which was originally investigated as an anxiolytic agent, in the treatment of depression. Since the randomized, controlled trials performed with Silexan have consistently used the HAMA as a primary outcome measure and have thus assessed depressed mood as a co-morbidity symptom, we performed a meta-analysis of these trials with focus on the effect of Silexan on co-occurring depressive symptoms in patients suffering from subthreshold anxiety and anxiety disorders.

## Methods

### Included trials

Until the end of the year 2020, five 10-week, randomized, double-blind, placebo-controlled clinical trials investigating Silexan in subthreshold anxiety and in anxiety disorders were completed with sponsorship of the manufacturer [41, 47–50]. We performed free-text searches of all fields of PubMed as well as of the European Union (EU) Clinical Trials Register, the International Standard Randomized Controlled Trial Number (ISRCTN) Registry and of the ClinicalTrials.gov registry to identify any additional trials with Silexan in patients with anxiety disorders. Search terms were ‘anxiety’ in combination with either ‘Silexan’, ‘Lasea’, ‘WS1265’ or ‘WS 1265’ (‘WS 1265’ was the internal code used by the manufacturer for Silexan) and suppressing the automatic PubMed translation of ‘Silexan’ to ‘lavender oil’ when building the search query. The literature from the earliest record until 30 December 2020 was covered.

### Interventions

Trials were eligible if participants received monotherapy with Silexan 1 × 80 mg/day as immediate-release soft gelatin capsules or a matching placebo for 10 weeks. Silexan is an essential oil manufactured from *Lavandula angustifolia* flowers by steam distillation that complies with the monograph Lavender oil of the European Pharmacopoeia and exceeds the quality requirements of the monograph. Batch to batch consistency is assured by a well-defined, standardized manufacturing process.

Analyses were performed on study participants who received either the recommended daily dose of the marketed product, i. e., 1 × 80 mg Silexan, or placebo. Results of treatment groups including active controls or Silexan administered at daily doses other than 80 mg/day were not considered in our meta-analysis.

### Meta-analysis outcomes

The present meta-analysis was conducted according to a prospectively defined analysis plan. The mean change from baseline to the individual end of treatment in the HAMA item ‘depressed mood’ defined as ‘loss of interest, lack of pleasure in hobbies, depression, early waking, diurnal swing’ and assessed by means of a five-point verbal rating scale ranging from 0 (‘not present’) to 4 points (‘very severe’) was compared between the treatment groups.

Moreover, the effect of Silexan on co-morbid depression/depressive symptoms in subthreshold anxiety and anxiety disorders was assessed by comparing mean changes from baseline of the total score of the HAMD or of the Montgomery Åsberg Depression Rating Scale (MADRS), if available, with the HAMD being the first choice in cases, where both scales were used. Furthermore, the mean change in the self-rated Hospital Anxiety and Depression Scale (HADS) [51] and the brief, observer-rated Raskin Depression Rating Scale (RDRS) [52] served as additional outcomes for the assessment of depressive symptoms.

For those cases in which the protocols did not require patients to be suffering from co-occurring depressive symptoms, we also performed a subgroup analysis for HAMA item ‘depressed mood’ that included only patients who presented with a score of at least 2 points (‘moderate’) at baseline. This cutoff was chosen in accordance with Kasper et al. [50], who used the same minimum score as an inclusion criterion in their trial for assuring that patients were suffering from comorbid subthreshold depression. For the subgroup analysis on the depression rating scales, we used cutoff scores of ≥ 7 points for the MADRS and of ≥ 8 points for the HAMD total scores that have been found to be indicative of at least mild depression [53, 54].

### Statistical methods

We performed a patient-level meta-analysis. The applicable analysis data set comprised the full analysis set (FAS) of the original protocols. For comparability with the published trial results, missing data were imputed by carrying forward the last valid observation.

To characterize the study populations, descriptive statistics were computed for age, sex, and premature withdrawal rate. The meta-analysis was based on a two-stage approach [55, 56]: within each trial, meta-analysis outcomes were analyzed using analysis of covariance (ANCOVA) with the difference between baseline and end of treatment for the outcome of interest as the dependent variable, treatment as a factor, and the baseline value of the analyzed outcome as a covariate. Marginal (adjusted) mean values and their standard deviations were then used as input for a random-effects meta-analysis on the treatment group mean value difference. Inverse variance weighting was used for combining the results of the single trials, and the DerSimonian–Laird method was applied for calculating the variance between the trials. As effect sizes, mean differences (MD) were calculated for the change of HAMA item ‘depressed mood’ and standardized mean differences (SMD) using Hedges’ g with bias correction for HAMD/MADRS total score changes. All *p* values are two-sided; values ≤ 0.05 were considered descriptively significant.

Heterogeneity between the trials was assessed using the *I*^2^ statistic in accordance with the criteria proposed by Deeks, et al. [57].

This meta-analysis was computed with R software (versions 3.1.2 and 3.6.0) using functions ‘metacont’ and ‘forest’ included in package meta (versions 4.3–2 and 4.13–0). All other analyses were performed in SAS statistical software version 9.4 for Windows.

## Results

### Characteristics of included trials

Searching PubMed resulted in 31 matches, none of which referred to a double-blind, randomized, placebo-controlled, therapeutic clinical trial in patients with subthreshold anxiety and anxiety disorders beyond those already mentioned. Searches in the indicated trial registers also did not add any clinical trials meeting these criteria.

The five trials included into our analysis were performed according to essentially similar protocols that differed mainly in the diagnosis for inclusion and in the derived inclusion and exclusion criteria as well as in some secondary outcome measures (Table 1). All trials have been approved by the appropriate ethics committee and have, therefore, been performed in accordance with the ethical standards laid down in the 1964 Declaration of Helsinki and its later amendments. Trial A [48], trial B [47] and trial C [50] assessed patients with subthreshold anxiety, and trial D [41] as well as trial E [49] investigated patients suffering from GAD. In all trials, the participants were male or female outpatients between 18 and 65 years of age and treated by a psychiatrist or by a general practitioner. In addition to meeting the diagnostic criteria for the diagnoses for inclusion shown in Table 1, eligible participants had to have a baseline HAMA total score ≥ 18 points and had to meet other anxiety specific eligibility criteria as shown in Table 1. In trials D and E, the HAMD was administered mainly for excluding patients suffering from MDD as primary diagnosis. All participants of trial C had to be suffering from comorbid, subthreshold anxiety and depression in accordance with the diagnosis for inclusion.

**Table 1:** Main study design characteristics and subject inclusion criteria

Trial	A [48]	B [47]	C [50]	D [41]	E [49]
Design characteristics	Double-blind, randomized, placebo-controlled, multicenter, parallel-group				
Diagnosis for inclusion	Anxiety disorder not otherwise specified (DSM-IV 300.00; ICD-10 F41.9)	Restlessness and agitation (ICD-10 R45.1)	Mixed anxiety and depressive disorder (ICD-10 F41.2)	Generalized anxiety disorder (DSM-IV 300.02, also corresponding to DSM-5 criteria; ICD 10 F41.1)	
Anxiety specific selection criteria	HAMA total score ≥ 18 points; HAMA items ‘Anxious mood’ and ‘Insomnia’ ≥ 2 points	HAMA total score ≥ 18 points; HAMA items ‘Tension’ and ‘Insomnia’ ≥ 2 points	HAMA total score ≥ 18 points; HAMA item ‘Anxious mood’ ≥ 2 points	HAMA total score ≥ 18 points; HAMA items ‘Anxious mood’ and ‘Tension’ ≥ 2 points; CAS total score ≥ 9 points	HAMA total score ≥ 18 points; HAMA items ‘Anxious mood’ and ‘Tension’ ≥ 2 points; HAMA sub-score ‘Psychic anxiety’ ≤ 21 points; CAS total score ≥ 9 points
Depression specific selection criteria	None	None	HAMA item ‘depressed mood’ ≥ 2 points	HAMD total score ≤ 17 points; HAMD item ‘Suicide’ < 2 points; RDRS total score ≤ 7 points	HAMD total score ≤ 17 points; HAMD items ‘depressed mood’ and ‘Suicide’ < 2 points; RDRS total score ≤ 7 points
Interventions	1 × 80 mg/day Silexan or placebo*, 10 weeks				
Primary efficacy outcome measures	HAMA total score change between baseline and end of treatment; trial C only: MADRS total score change between baseline and end of treatment				

*HAMA* Hamilton Anxiety Rating Scale [12], *HAMD* Hamilton Depression Rating Scale [13], *CAS* Covi Anxiety Scale [65], *RDRS* Raskin Depression Rating Scale [52], *MADRS* Montgomery Åsberg Depression Rating Scale [66], *ICD-10* International Classification of Diseases, 10th revision, *DSM-IV* Diagnostic and Statistical Manual of Mental Disorders, 4th edition, *DSM-5* Diagnostic and Statistical Manual of Mental Disorders, 5th edition

*In addition to Silexan 80 mg/day, trial D included treatment groups receiving Silexan 10 or 40 mg/day, and trial E included groups that received Silexan 160 mg/day or paroxetine. Results for Silexan other than those for the marketed dosage of 80 mg/day or for active comparators are not covered in this work

The schedule of each trial started with a 3–7-day qualification phase after which eligible patients were randomized to receive Silexan or placebo for 10 weeks. Eligibility criteria had to be met both at the start (screening) and at the end (baseline) of the qualification phase. In trials A, B, D, and E, patients were not required to be suffering from co-occurring depressive symptoms. In trials A, B, D, and E, post-baseline outcome assessments were scheduled every 2 weeks, while the protocol of trial C included assessments at the end of weeks 1, 2, 4, 7, and 10.

Study participants received either Silexan 1 × 80 mg/day as immediate-release soft gelatin capsules or a matching placebo for 10 weeks. Trial D was a dose-finding trial that also included treatment arms with 10 and 40 mg/day Silexan. In trial E, paroxetine served as an active control, and another group received Silexan 160 mg/day.

For trials C, D, and E, the effect of Silexan on comorbid depression or on co-occurring depressive symptoms could be assessed based on the change of the total score of the HAMD (trials D and E) or the MADRS (trial C) between baseline and individual end of treatment. The total scores of the MADRS and the HAMD observer-rated depression scales served as the main instruments for assessing severity of depression according to the original protocols of trials C, D, and E. In trials D and E, HAMD assessments were obtained at baseline as well as at weeks 4 and 10 of randomized treatment. In trial C, the MADRS was administered at baseline and at all post-baseline visits. Moreover, the self-rated Hospital Anxiety and Depression Scale [HADS; 60] was used in trial C, and the brief, observer-rated Raskin Depression Rating Scale [RDRS; 44] was administered in trials D and E as additional secondary outcomes for the assessment of depression.

### Characteristics of trial participants

In the pooled data set, a total of 1213 patients (Silexan *N* = 610; placebo *N* = 603) had been randomized and 1172 (Silexan *N* = 587; placebo *N* = 585) had been analyzed for efficacy in the FAS of the underlying five trials (Table 2). Since levels of depression tended to decrease during the randomized treatment period (see details below) and missing data (mainly resulting from premature withdrawal) were imputed by carrying the last observed value forward, premature withdrawal might have caused some bias of the depression scale results against Silexan.

**Table 2:** Study population baseline characteristics based on full analysis set (number and % or mean ± SD)

Trial	Treatment	Randomized	Drop-outs	FAS	Females	Age (years)	HAMA ‘depressed mood’
A	Silexan	110	18 (16.4%)	104	73.1%	45.6 ± 11.4	2.1 ± 0.8
	Placebo	111	14 (12.6%)	108	76.9%	46.6 ± 11.3	2.2 ± 0.9
B	Silexan	86	12 (14.0%)	86	72.1%	48.0 ± 11.3	1.9 ± 0.8
	Placebo	84	10 (11.9%)	84	71.4%	46.9 ± 12.7	2.0 ± 0.9
C	Silexan	160	15 (9.4%)	159	66.0%	47.7 ± 12.6	2.5 ± 0.5
	Placebo	158	13 (8.2%)	156	72.4%	47.9 ± 12.6	2.5 ± 0.6
D	Silexan	118	11 (9.3%)	103	76.7%	43.3 ± 11.7	1.0 ± 0.7
	Placebo	113	8 (7.1%)	102	65.7%	45.5 ± 11.5	1.2 ± 0.7
E	Silexan	136	17 (12.5%)	135	70.4%	45.7 ± 11.5	1.2 ± 0.8
	Placebo	137	19 (13.9%)	135	73.3%	44.6 ± 12.3	1.0 ± 0.7
Pooled	Silexan	610	73 (12.0%)	587	71.0%	46.1 ± 11.9	1.8 ± 0.9
	Placebo	603	64 (10.6%)	585	72.1%	46.4 ± 12.2	1.8 ± 1.0

*FAS* full analysis set, *HAMA* Hamilton Anxiety Rating Scale, *n/a* not applicable, *SD* standard deviation

The study participants’ age averaged around 46 years. More than ^2^/_3_ of the patients of all trials were female.

Within each trial, the baseline treatment group mean values for the HAMA item ‘depressed mood’ did not differ significantly (never exceeding 2.5 points). Baseline scores were highest in trial C performed in MADD, which was the only trial that included only patients with comorbid subthreshold depression at baseline, and lowest in the GAD trials D and E, both of which explicitly excluded patients with more severe depression. In trials C through E, the baseline total scores of the MADRS and the HAMD also support the baseline comparability of the treatment groups regarding their average severity of depressive symptoms (Table 3). Moreover, the baseline treatment group mean values for the MADRS (trial C) and for the HAMD total score (trials D and E) were in a range typically found in patients with mild to moderate intensity of depression [53, 54].

**Table 3:** Depression scales total score—baseline value and intraindividual change between baseline and treatment end (sample size, mean ± SD, *p* values for treatment group comparisons)

Analysis set	Trial	Scale	Assessment^#^	Silexan	Placebo	*p*^$^
FAS	C	MADRS	Baseline	(159) 22.0 ± 6.4	(156) 22.1 ± 6.1	
			Change	(159) − 9.2 ± 9.9	(156) − 6.1 ± 7.6	< 0.01
	D	HAMD	Baseline	(103) 11.4 ± 3.0	(102) 11.6 ± 2.9	
			Change	(102) − 4.5 ± 4.2	(101) − 3.7 ± 4.5	0.20
	E	HAMD	Baseline	(135) 11.7 ± 3.2	(135) 11.8 ± 2.9	
			Change	(133) − 4.1 ± 5.0	(134) − 2.8 ± 4.7	0.02
Depression subset*	C	MADRS	Baseline	(159) 22.0 ± 6.4	(156) 22.1 ± 6.1	
			Change	(159) − 9.2 ± 9.9	(156) − 6.1 ± 7.6	< 0.01
	D	HAMD	Baseline	(94) 11.9 ± 2.6	(93) 12.1 ± 2.4	
			Change	(93) − 4.7 ± 4.2	(92) − 3.8 ± 4.6	0.16
	E	HAMD	Baseline	(116) 12.5 ± 2.6	(122) 12.4 ± 2.3	
			Change	(114) − 4.6 ± 5.1	(121) − 2.8 ± 4.8	0.01

*FAS* Full analysis set, *MADRS* Montgomery Åsberg Depression Rating Scale, *HAMD* Hamilton Depression Rating Scale, *SD* standard deviation

*Trial C: baseline MADRS total score ≥ 7 points; trials D, E: baseline HAMD total score ≥ 8 points

^#^Intraindividual change: end of randomized treatment—baseline value

^$^*p* value from ANCOVA with factor treatment and baseline value as covariate

### HAMA item ‘depressed mood’

**Fig. 1:**
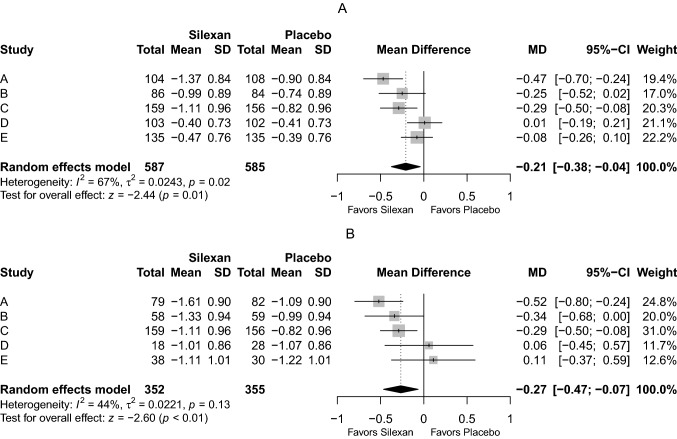
Meta-analysis of Hamilton Anxiety Rating Scale item ‘depressed mood’, intraindividual change between baseline and end of treatment (last observation carried forward). Panel **A**: all patients in the full analysis set; Panel **B**: patients with a baseline score ≥ 2 points

With respect to the pooled mean reduction in the outcome HAMA item ‘depressed mood’ between baseline and treatment end, we found a significant superiority of Silexan over placebo (MD = − 0.21, 95% confidence interval; CI − 0.38 to − 0.04; *p* = 0.01) (Fig. 1 Panel A). In the subset of patients with a baseline score ≥ 2 points (i. e., those who had at least moderate depressive symptoms at baseline, including all patients from trial C, where this was an inclusion criterion), the overall meta-analysis effect of Silexan was even more pronounced than in the complete FAS (MD = − 0.27, 95% CI − 0.47 to − 0.07; *p* < 0.01) (Fig. 1 Panel B).

With *I*^2^ = 67% for the FAS and *I*^2^ = 44% for the subset with at least moderate depressive symptoms at baseline, Fig. 1 also indicates substantial heterogeneity between the trials. This was mainly attributable to the fact that the participants of trials D and E, who had substantially lower depression scores at baseline, due to the exclusion criteria in these trials (Tables 1, 2), showed lower absolute score reductions during randomized treatment, and thus also smaller absolute treatment group differences.

**Fig. 2:**
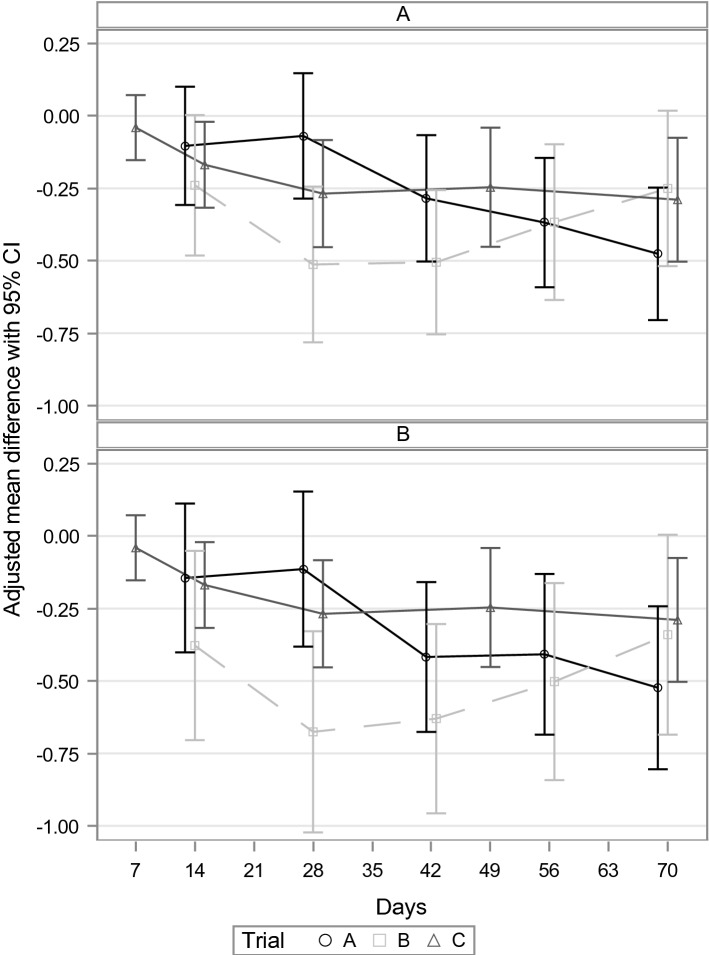
Hamilton Anxiety Rating Scale item ‘depressed Mood’ change from baseline—mean value differences between Silexan and placebo for all patients in the full analysis set (Panel **A**) and for patients with a baseline score ≥ 2 points (Panel **B**; negative values favor Silexan)

For the trials performed in patients with subthreshold anxiety, Fig. 2 shows the mean value differences between Silexan and placebo (including the associated 95% Cis) for HAMA item ‘depressed mood’. Descriptively significant advantages for Silexan were observed from day 42 of randomized treatment in trial A, between day 14 (patients with a baseline score ≥ 2 points) or day 28 (FAS) and day 56 in trial B, and from day 14 in trial C (*p* ≤ 0.05). In trials B and C, the stabilization or decrease of the difference between Silexan and placebo after day 28 was attributable to an increasingly large placebo effect, while the scores in the Silexan group stabilized (trial B) or decreased at a slower rate than during the initial weeks of randomized treatment (trial C).

### MADRS and HAMD

**Fig. 3:**
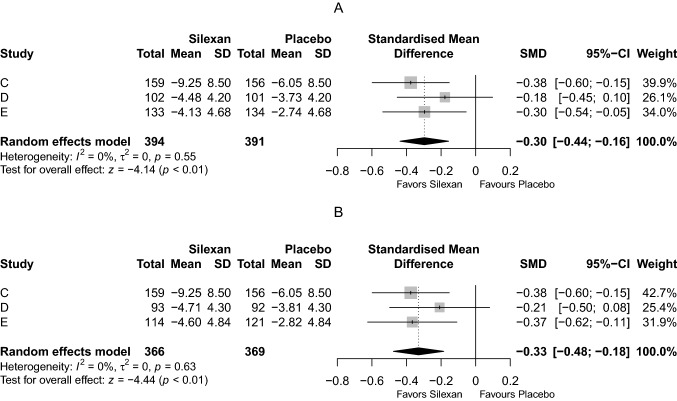
Meta-analysis of depression rating scales total score, intraindividual change between baseline and end of treatment (trial C: Montgomery Åsberg Depression Rating Scale; trials D, E: Hamilton Anxiety Rating Scale; SMD: standardized mean value difference; last observation carried forward). Panel **A**: all patients in the full analysis set; Panel **B**: patients with at least mild depression at baseline

The mean total score reduction in MADRS/HAMD was significantly higher in the pooled Silexan group compared with the placebo group (SMD = − 0.3, 95% CI − 0.44 to − 0.16; *p* < 0.01) (Table 3), with minimal heterogeneity between the trials (*I*^2^ = 0%; Fig. 3).

As all patients in trial C and the majority of participants of trials D and E had at least mild symptoms of depression (i. e., a MADRS total score ≥ 7 or a HAMD total score ≥ 8) at baseline, the results in the FAS and in the ‘Depression’ subset were similar, with slightly larger effect sizes favoring Silexan in trials D and E as well as overall in the ‘Depression’ subset.

### Other depression scales (full analysis set)

In trial C, average intraindividual decreases by 2.2 ± 5.0 (mean ± SD) and by 1.8 ± 4.1 points were observed in the self-rated HADS between baseline and end of treatment (*p* = 0.52) for Silexan and placebo, respectively, for the depression sub-score, following baseline values of 10.7 ± 4.7 and of 10.5 ± 4.2 points.

In trials D and E, the RDRS was mainly used as an additional secondary outcome for the assessment of depression to assure the exclusion of patients suffering from a MDE as primary diagnosis. In trial D, the RDRS total score in the Silexan group decreased by 0.8 ± 1.5 points from a baseline average of 5.2 ± 1.1 points, compared to a baseline mean value of 5.1 ± 1.1 points and a decrease by 0.5 ± 1.4 points in the placebo group (*p* = 0.14). In trial E, the patients in the Silexan 80 mg/day group showed a RDRS baseline mean value of 5.1 ± 1.1 points and a decrease between baseline and end of treatment by 0.7 ± 1.6 points, compared to a baseline value of 5.2 ± 1.1 points and a decrease by 0.4 ± 1.6 points for placebo (*p* = 0.17).

## Discussion

This meta-analysis of five double-blind, randomized, placebo-controlled trials in subthreshold anxiety and anxiety disorders found that Silexan, which has already been demonstrated to be an efficacious anxiolytic compound [31, 39], is also effective in reducing co-occurring depressive symptoms.

In the trials in subthreshold anxiety disorder in which no specific depression scale was administered, Silexan was associated with a reduction of the treatment group mean value for depressed mood at or above 50% of the baseline value, notably in patients with at least moderate symptoms of depression at baseline, with significant superiority over placebo in two trials and a borderline significant result in the third, based on HAMA item ‘depressed mood’.

In the only trial investigating patients with MADD, where comorbid subthreshold depression at baseline was required as a part of the clinical diagnosis for inclusion, a significant antidepressant effect of Silexan over placebo was observed for the MADRS total score already after 4-week randomized treatment, and the treatment group difference remained significant until the end of the trial after 10 weeks.

For the two trials in GAD, which explicitly excluded patients with more severe depression, one needs to consider that patients were only eligible for inclusion if they presented with a HAMD total score ≤ 17 points and with a score < 2 points for HAMA item ‘depressed mood’ at both screening and baseline, which resulted in study populations with comparatively low rates for comorbid depressive symptoms. It is, therefore, not surprising that a single-item measure such as HAMA item ‘depressed mood’, with its limited sensitivity for change over time, did not capture a meaningful antidepressant effect in this specific patient population. By contrast, a clear antidepressant effect of Silexan over placebo was observed for HAMD total score change, with significant superiority in one of the two GAD trials even though only patients with predominantly mild depressive symptoms were included.

In summary, our meta-analysis indicates that co-occurring depressive symptoms improved significantly during treatment with Silexan. This observation is consistent with the existing psychopharmacotherapeutic evidence supporting a possible direct antidepressant effect of the herbal medicinal product [35, 37]. In vitro, Silexan was shown to improve synaptic neuroplasticity, which is discussed as a common pathway for the mechanisms of action of most antidepressants [58, 59]. Similarly to previous observations in antidepressants, a significant effect on neurite outgrowth in PC12 cells and on synapse density in primary hippocampal neurons has been assumed for Silexan [43]. In vivo, Friedland, et al. [43] performed a forced swimming test in rats, a behavioral model commonly used to assess activity of antidepressant therapies and found that the effects of Silexan were comparable to those of imipramine that served as an active control. Moreover, linalool, one of the major constituents of Silexan, was found to show antidepressant-like properties in an immobilization test performed in mice [60]. Whether the alleviation of depressive symptoms could be mediated by the anxiolytic effect of Silexan, might be subject to further investigation.

The abovementioned results and consequent assumptions on beneficial and clinically meaningful effects of Silexan on both, anxious and depressive symptoms, might be further underlined by findings derived from clinical trials reporting superior effects of Silexan on sleep disturbances, psychomotor agitation and somatic symptoms including fatigue and pain for instance, which represent frequent and burdensome manifestations occurring in the course of both clinical phenotypes [44–46]. It might be noteworthy in this regard that the recently published meta-analysis focusing on Silexan effects on somatic symptoms and physical health in general was conducted in a patient population that is identical with that investigated in the present meta-analysis, whereby a similar approach using HAMA items to evaluate the respective target-symptoms was employed [46].

As was already shown previously, Silexan is well tolerated and does not cause pharmacological interactions or withdrawal symptoms at daily doses of 80 or 160 mg [39]. A good tolerability of psychopharmacotherapy with Silexan can also be assumed as a result of our findings, which show a pooled drop-out rate of 12.0% detected for Silexan compared to 10.6% for placebo (Table 2).

While no clinical trials with Silexan in patients with primary MDD have been completed yet, it is a strength of this investigation that our analyses cover all randomized, placebo-controlled trials performed with the herbal product in subthreshold anxiety and anxiety disorders, representing the complete existing body of evidence for the effect of Silexan on co-occurring depressive symptoms. A limitation of our analyses could be the fact that the assessment of the antidepressant effect in two of the five trials (A and B) had to rely solely on a single item from the HAMA questionnaire. In trial C performed in MADD, the results obtained for this item were consistent with those for the MADRS. However, in contrast to the results obtained for the HAMD, the single-item measure apparently lacked the sensitivity for monitoring intraindividual change of depressive symptom intensity in the at most mildly depressed patients of trials D and E performed in GAD. In summary, the resultant heterogeneous clinical manifestations of comorbid depressive symptoms in patients with primary (subthreshold) anxiety disorders might explain the subtle differences in the observed antidepressant effects and should be considered while interpreting the present results.

Our analyses also reveal that patients with more severe depressive symptoms at baseline tended to show more pronounced symptom alleviation during treatment with Silexan. Since not all trial participants showed substantial symptoms of depression, the analyses based on the FAS of the studies may have underestimated the true antidepressant effect of Silexan.

Finally, it should be considered that the present work is based on data which were gathered and published by authors of the same research group who are largely represented in this and a further recently published meta-analysis [46]. The detected effect sizes might, hence, exhibit potentially higher similarities than it would be the case, when studies of different research groups would be involved, which may result from the way how the distinct parameters were analyzed, how the patients were recruited and sampled, and how the data were assessed by the study interviewers [61, 62]. A specific example of the latter phenomenon represented by a similar network meta-analysis including 4 papers published by Kasper et al. [63] has already been discussed in the aforementioned meta-analysis, highlighting that all included studies were performed in accordance with Good Clinical and Scientific Practice [46]. Hence, the data and the reported results should be considered robust and scientifically sound.

In conclusion, the results of our meta-analysis underline that Silexan, at the marketed dosage of 1 × 80 mg/day, has a significant alleviating effect on co-occurring depressive symptoms in patients suffering from subthreshold anxiety and an anxiety disorder. While our analysis does not provide conclusive evidence as to whether this is a direct antidepressant effect or an effect mediated by the anxiolytic activity of the compound, evidence from in-vitro and in-vivo pharmacological experiments as well as information about Silexan’s mechanism of action could explain a direct antidepressant effect that may result from an improvement of neuroplasticity and its effects on monoaminergic neurotransmission [37, 38, 40, 43, 64]. Taken together, the results thus indicate that Silexan reduces depressive symptoms in anxiety patients and, in addition, might have a beneficial effect in patients with depressive disorders. This should be confirmed in future trials.

## Data Availability

Data sharing is not applicable to this article as no new data were created or analyzed in this study.

## Footnotes

1 Silexan® is the active substance of Lasea® (Dr. Willmar Schwabe GmbH & Co. KG, Karlsruhe, Germany).
